# Modulation of the ACOD1/itaconate pathway differentially affects atherosclerosis severity across genetic models and sexes

**DOI:** 10.1172/JCI182472

**Published:** 2025-06-12

**Authors:** Lara Haase, Anouar Belkacemi, Laura Neises, Nicole Kiweler, Christine Wesely, Rosanna Huchzermeier, Maja Bozic, Arefeh Khakdan, Marta Sánchez, Arnaud Mary, Nadja Sachs, Hanna Winter, Enrico Glaab, Michael T. Heneka, Emiel P.C. van der Vorst, Michel Mittelbronn, Johannes Meiser, Jochen G. Schneider

**Affiliations:** 1 Luxembourg Centre for Systems Biomedicine, University of Luxembourg, Campus Belval, Esch-sur-Alzette, Luxembourg.; 2 Cancer Metabolism Group, Department of Cancer Research, Luxembourg Institute of Health, Luxembourg, Luxembourg.; 3 Institute of Pharmacology, Heidelberg University, Heidelberg, Germany.; 4 German Centre for Cardiovascular Research (DZHK), Heidelberg/Mannheim/Munich, Germany.; 5 Experimental and Clinical Pharmacology and Toxicology, Saarland University, Homburg, Germany.; 6 Department of Internal Medicine-I, University Hospital Aachen, Aachen, Germany.; 7 Department of Life Science and Medicine, University of Luxembourg, Campus Belval, Esch-sur-Alzette, Luxembourg.; 8 Department of Vascular Surgery, and; 9 Institute of Molecular Vascular Medicine, TUM University Hospital, Munich, Germany.; 10 Institute for Cardiovascular Prevention, Ludwig-Maximilians-Universität, Munich, Germany.

**Keywords:** Cardiology, Vascular biology, Atherosclerosis, Macrophages

**To the Editor:** Itaconate is a macrophage-derived metabolite central to immunometabolism (1, 2). Its role, including 4-octyl-itaconate (4-OI), has sparked interest in atherosclerosis because of its known inflammatory characteristics. Song et al. reported that 4-OI inhibits atherosclerosis in male mice in a nuclear factor erythroid 2–related factor 2–dependent (Nrf2-dependent) manner using an adeno-associated virus atherosclerosis model, while *cis*-aconitate decarboxylase (*Acod1*) knockdown, which reduces endogenous itaconate, aggravates atherosclerosis. The study suggests that “activation of the itaconate pathway may represent an important approach to treat atherosclerosis,” supporting the idea that 4-OI may have therapeutic potential (3). However, chemical differences from endogenous itaconate limit the relevance of 4-OI for modeling itaconate biology (2).

We assessed *Acod1* deficiency in *Ldlr^–/–^* and *Apoe^–/–^* mouse models of athersclerosis. In *Ldlr^–/–^* *Acod1^–/–^* mice (male and female), plaque size in the aortic root and thoracic and abdominal aorta matched control plaque size (Figure 1, A–E). Similar findings were identified in *Apoe^–/–^* mice, except female *Apoe^–/–^* *Acod1^–/–^* mice showed reduced aortic arch plaque size (Supplemental Figure 1, A–E; supplemental material available online with this article; https://doi.org/10.1172/JCI182472DS1). Pooling sexes revealed reduced plaque size in *Apoe^–/–^* *Acod1^–/–^* mice in the aortic arch (*P* = 0.0043) and thoracic and abdominal aorta (*P* = 0.0218), but not on the *Ldlr^–/–^* background.

Lesional foam cell area and collagen accumulation did not differ between genotypes in the *Apoe^–/–^* mouse model (Supplemental Figure 1, F and G), and heart weight was also comparable between genotypes in both mouse models (Supplemental Figure 1, H and I). These results align with Harber et al., who found more stable plaques in *Ldlr^–/–^* female mice transplanted with male *Acod1^–/–^* bone marrow (4).

Cholesterol influx and efflux in bone marrow–derived macrophages from *Ldlr^–/–^* *Acod1^–/–^* and *Apoe^–/–^* *Acod1^–/–^* mice were unchanged (Supplemental Figure 2, A–C). Itaconate is known to affect cytokines (1, 2). Unlike the observation made by Song et al. (3), we found no difference in plasma cytokines in atherosclerotic male mice (Supplemental Figure 2D). However, we identified a significant increase in the proportion of circulating T cells in female *Apoe^–/–^* *Acod1^–/–^* mice, which was not observed in *Ldlr^–/–^* *Acod1^–/–^* mice (Figure 1, F and G, and Supplemental Figure 2E).

Male *Ldlr^–/–^* *Acod1^–/–^* mice gained significantly more weight and had elevated plasma cholesterol (Figure 1, H and I), suggesting a metabolic shift in *Ldlr^–/–^* mice (5). Yet, hepatic cholesterol and triglyceride content did not differ between *Ldlr^–/–^Acod1^–/–^* and control mice (Supplemental Figure 2, H and I). Liver IL-1β increased in pooled samples but did not differ when compared by sex (Figure 1J and Supplemental Figure 2J). Body weight and plasma cholesterol did not differ between *Apoe^–/–^* *Acod1^–/–^* and control mice (Supplemental Figure 2, F and G).

Glucose tolerance tests after 4 weeks of Western diet feeding revealed no difference in *Ldlr^–/–^* *Acod1^–/–^* mice versus controls (Supplemental Figure 3, A and B). Consistent with these findings, hepatic and adipose p-AKT/AKT ratios were similar between genotypes (Supplemental Figure 3, C–F). There was a slight trend toward lower IRS-2–dependent PI3K activity in male *Ldlr^–/–^* *Acod1^–/–^* mice. The expression level of *Cpt1a*, *Cpt2*, *Acox1*, *Acox2*, *Acly*, *Srebf1*, *Ppara*, *Pparg*, and *Acat2* was not different between both genotypes (Supplemental Figure 3, G–I).

Cyr et al. (6) recently reported a protective role for itaconate in mice, employing the same disease model but different Western diet and bone marrow transplantation. However, their experiments included male and female mice, pooling results and not assessing sex-specific differences in atherosclerosis. While we observe similar differences when pooling all mice by sex, these effects vanish when data are analyzed separately by sex (Figure 1, A–E). Consistent with Song et al., Cyr et al. also reported *ACOD1* upregulation in human coronary atherosclerotic lesions, suggesting a protective role for itaconate.

RNA-seq and single-cell RNA-seq (scRNA-seq) data revealed low *ACOD1* expression in human carotid plaques, in contrast with established atherosclerosis markers. In scRNA-seq data from early and advanced human carotid lesions, *ACOD1* was detected in only approximately 0.75%–1% of resident macrophages from male individuals and low expression was confirmed in macrophages from asymptomatic females (Supplemental Figure 4, A–G), suggesting sex- and symptom-related differences. Nevertheless, immunohistochemistry confirmed ACOD1 expression associated with inflammatory cells of plaques from both sexes (Supplemental Figure 4H). Although these findings confirm the presence of ACOD1 in atherosclerotic lesions, low expression levels likely contribute to the variability of the data.

In conclusion, modulation of the ACOD1/itaconate pathway varies by the model used and the sex of the species. The relevance of ACOD1 in human atherosclerosis warrants further studies.

## Supplementary Material

Supplemental data

Supporting data values

## Figures and Tables

**Figure 1:**
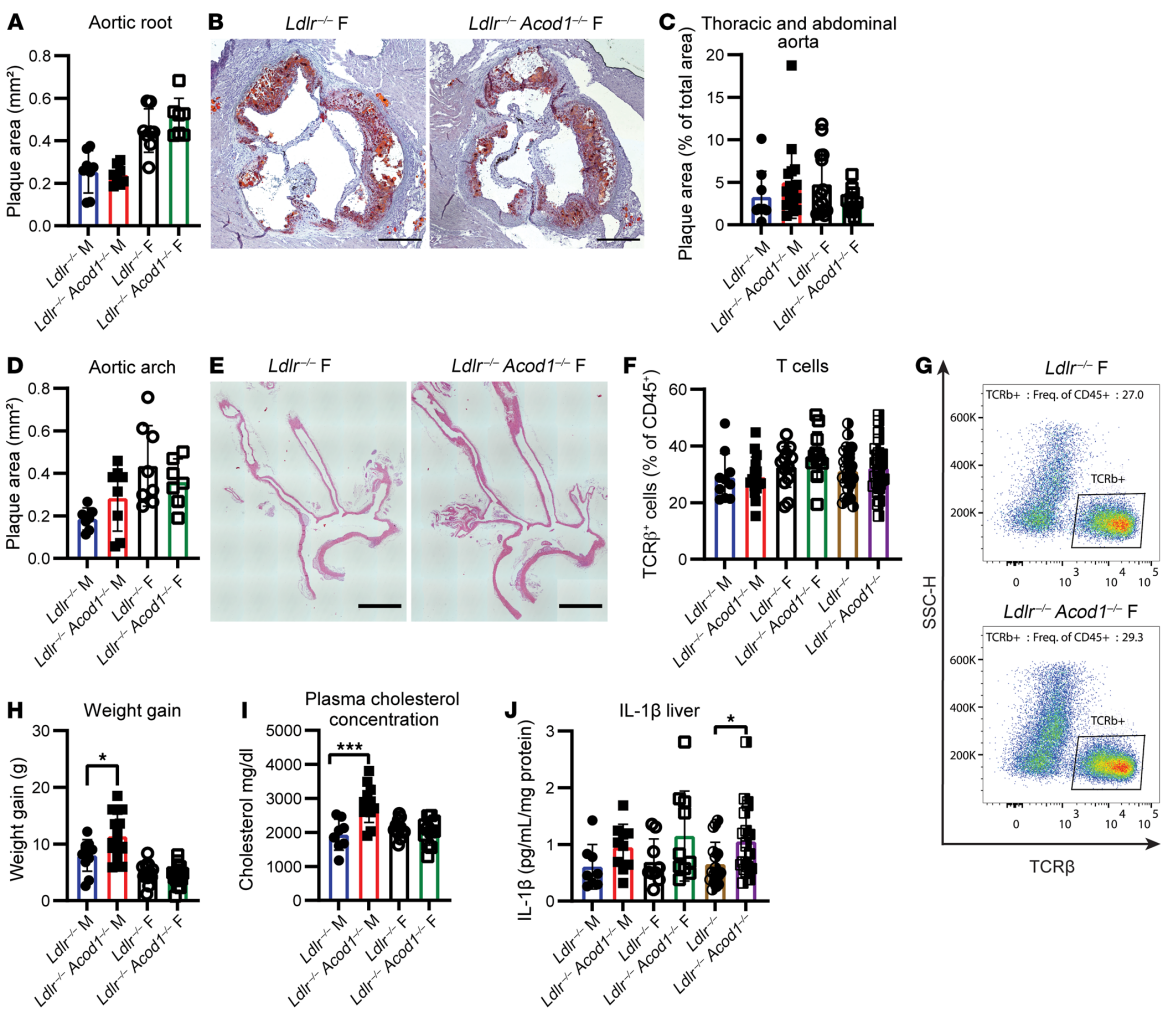
*Acod1* deletion has no effect on atherosclerosis but shows sex-dependent cholesterol metabolism in the Ldlr model. *Ldlr*^–/–^*Acod1^–/–^* and control *Ldlr^–/–^* mice (6–12 weeks old) were fed a Western diet (Envigo, TD-88137) for 12 weeks. Plaque area was measured in the aortic root (**A**, *n* = 7–8/sex/group), thoracic/abdominal aorta (**C**, males *n* = 9–16, females *n* = 12–14), and aortic arch (**D**, *n* = 7–8/sex/group). Representative aortic arch images from females are shown (**B** and **E**). Circulating T cells were analyzed by flow cytometry (**F**, males *n* = 10–16, females *n* = 12–14, pooled right), gated as CD45^+^TCRβ^+^CD19^–^ cells. Shown are representative dot plots (**G**). Weight gain (**H**, males *n* = 11–16, females *n* = 14–15), plasma cholesterol (**I**, *n* = 8–11/group), and liver IL-1β levels (**J**, *n* = 8–10/group, pooled right) were quantified. Data are shown as mean ± SD. *P < 0.05; ***P < 0.001 by Mann-Whitney *U* test (**A**, **C**, and **D**) or Welch’s *t* test (**F**–**J**). Scale bars: 300 μm (**B**) and 1 mm (**E**).

## Version 1 06/12/2025

In-Press Preview

## Version 2 08/01/2025

Electronic publication
