# Supplementary material for: Modulation of the ACOD1/itaconate pathway differentially affects atherosclerosis severity across genetic models and sexes
Source: J Clin Invest. 2025 Jun 12;135(15):e182472. doi: 10.1172/JCI182472 (PMC12324055; doi:10.1172/JCI182472)
Supplement: Supplemental data [file jci-135-182472-s002.pdf]

Modulation of the Acod1/itaconate pathway differentially affects atherosclerosis severity across genetic models and sexes

Lara Haase[1,2*], Anouar Belkacemi[3,4*], Laura Neises[2], Nicole Kiweler[2], Christine Wesely[5], Rosanna Huchzermeier[6], Maja Bozic[7], Arefeh Khakdan[7], Marta Sánchez[7], Arnaud Mary[1], Nadja Sachs[4,8], Hanna Winter[4,9], Enrico Glaab[1], Michael T. Heneka[1], Emiel P.C. van der Vorst[6,10], Michel Mittelbronn[7], Johannes Meiser[2*], Jochen G. Schneider[1,7*]

1. Luxembourg Centre for Systems Biomedicine, University of Luxembourg, Campus Belval, Esch-sur-Alzette, Luxembourg
2. Cancer Metabolism Group, Department of Cancer Research, Luxembourg Institute of Health, Luxembourg, Luxembourg
3. Institute of Pharmacology, Heidelberg University, Heidelberg
4. German Centre for Cardiovascular Research (DZHK), Heidelberg/Mannheim/Munich, Germany.
5. Experimental and Clinical Pharmacology and Toxicology, Saarland University, Homburg, Germany.
6. Department of Internal Medicine-I, University Hospital Aachen, Germany.
7. Department of Life Science and Medicine, University of Luxembourg, Campus Belval, Esch-sur-Alzette, Luxembourg
8. Department of Vascular Surgery, TUM University Hospital Munich, Germany
9. Institute of Molecular Vascular Medicine, TUM University Hospital, Munich, Germany
10. Institute for Cardiovascular Prevention, Ludwig-Maximilians-Universität, Munich, Germany

[*]Contributed equally

Corresponding author:

Jochen G. Schneider

Luxembourg Centre for Systems Biomedicine,
University of Luxembourg, Campus Belval,
7, avenue du Swing,
L-4362-Esch-sur-Alzette, Luxembourg

Email: jochen.schneider@uni.lu
Phone: +3524666446154

Conflict of interest: The authors declare no conflict of interest.

Supplemental Methods and Figures

Supplemental methods

Animals. The *Acod1*$^{-/-}$ mice were generated by Dr. Haruhiko Koseki at the RIKEN Institute (Yokohama, Japan) using stem cells obtained from the Knockout Mouse Project Repository (KOMP) under the strain ID: Irg1$^{tm1a(KOMP)Wtsi}$. The generated mice are on the C57BL/6N genetic background. The *Apoe*$^{-/-}$ and *Ldlr*$^{-/-}$ mice (C57BL/6J) were purchased from the Jackson Laboratory, strain number: 002052 and 002207 respectively. For the atherosclerosis experiments we used *Apoe*$^{-/-}$*Acod1*$^{-/-}$ and *Ldlr*$^{-/-}$*Acod1*$^{-/-}$ as knock out mice while *Apoe*$^{-/-}$ *Acod1*$^{+/+}$ and *Ldlr*$^{-/-}$ *Acod1*$^{+/+}$ littermates were used as wild-type control. The generated double knock-out mice and littermates are on the C57BL/6NJ genetic background. The genotype of the mice was confirmed by polymerase chain reaction (PCR) prior to experimentation.

Atherosclerosis induction. To induce atherosclerosis, 6 to 12 weeks old *Apoe*$^{-/-}$ or *Ldlr*$^{-/-}$ animals were given a western type diet for 12 weeks (Envigo, TD.88137). They were then sacrificed with an overdose of isoflurane (Baxter) inhalation followed by blood collection from the vena cava. The blood was placed in an Ethylenediaminetetraacetic acid (EDTA) coated tube (Sarstedt, Microvette) for flow cytometry and plasma collection. Directly after blood collection, the mice were perfused with 10mL PBS (Westburg, LO BE17-515Q). The liver was removed and snap frozen for further analysis. The aorta and heart were carefully removed and placed in 4% formol (VWR chemicals, 11699404) for 48h for histological analyses. The plasma cholesterol levels were measured using a cholesterol quantitation kit (Sigma Aldrich, MAK043) and cytokines were measured using the mouse atherosclerosis array C1 (RayBiotech, AAM-ATH-1-8) according to the manufacturers protocol.

Histology.

Aortic root: after fixation, the heart was placed in 15% w/v sucrose solution in PBS for 6h followed by a 30% w/v sucrose solution overnight. The tissue was then embedded in Tissue-Tek O.C.T Compound (Sakura, 4583) and snap frozen using isopentane and dry ice for cryosection. The embedded tissue was serially cut at 4µm on a cryostat (Leica CM1850 UV). Four sections were collected for each mouse with 40µm between each section. The sections were collected once the aortic root leaflets were visible. To detect lipids in plaques on cryosectionned tissue, the slides were incubated in an Oil-red-O solution (1.8mg/mL Oil-red-O Sigma, O0625-25G, in 60% v/v isopropanol, filtered before use). The slides were counterstained with a hematoxylin solution acc. to Gill II (Carl Roth, T864.2). To stain collagen on cryosectionned tissue, the slides were incubated in several solutions. Bouin's solution, Weigert's Iron Hematoxylin solution (Hematoxylin 5mg/mL, Ferric chloride 5mg/mL in 50% ethanol and 0.5% Acetic Acid), Biebrich Scarlet-Acid Fuchsin solution (Biebrich Scarlet 9mg/mL, Acid Fuchsin 1mg/mL in 1% Acetic Acid), Phosphomolybdic-Phosphotungstic solution (Phosphomolybdic acid, 12.5mg/mL, Phosphotungstic acid 12.5mg/mL), Aniline blue solution (Aniline Blue 25mg/mL in 4% acetic acid) and 1% acetic acid solution. The slides were finally dehydrated and mounted with Entellan (Sigma Aldrich, 1079600500)

Aortic arch: After fixation, the aortic arch was dehydrated with a tissue processor (Leica TP1020) for paraffin embedding. The embedded tissue was serially cut at 3µm on a microtome. Three sections were collected for each mouse with 36µm between each section. The sections were collected once the brachiocephalic artery, left common carotid artery and

left subclavian artery were visible. The paraffin-embedded sections were stained with hematoxylin and eosin (H&E) using a Ventana HE staining system (Roche).

The thoracic and abdominal aorta was prepared for the en face analysis. After fixation, the aorta was cut open from top to bottom. The adventitia was then carefully removed. During the entire process the aorta remained moist with regular PBS washes. The aorta was then washed in a 60% isopropanol solution and stained with an Oil-red-O solution (3mg/mL in 60% isopropanol, filtered).
Pictures were taken with a Nikon Eclipse Ni microscope using the NIS-Elements software (Nikon) and were analysed using ImageJ. The foam cell area was analysed with the H&E staining as they are stained with a light pink colour due to their high lipid content.

Immunohistochemistry of human carotid arteries. Samples of human carotid artery plaque were obtained during carotid endarterectomy (CEA), fixed in 2% paraformaldehyde at room temperature for 48 hours, paraffin-embedded, and sectioned into 2 μm slices. The material is part of the Munich Vascular Biobank (1). Tissue collection was performed in accordance with the Declaration of Helsinki and approved by the local ethics committee (Ethikkommission TU München: 2799/10). Based on histomorphological criteria, plaques were classified as stable or unstable/ruptured according to the American Heart Association (AHA) guidelines following Stary *et al.* (2) and fibrous cap thickness as described by Redgrave *et al*. (3). Transverse sections from ten individual plaques were selected for immunohistochemical analysis using anti-IRG1 (Abcam AB238580) and anti-CD68 (Dako M0814) antibodies. CD68 staining was performed using the Impress Polymer Kit (Vector Laboratories), and IRG1 staining with a two-step biotin-streptavidin kit (Bio SB). Control tissue was obtained from deceased organ donors without known cardiovascular disease (data not shown). High-resolution scans were analysed using QuPath software (4).

Flow Cytometry. The whole blood was lysed with ACK lysis buffer and washed with PBS prior to the live/dead staining. The Zombie Red solution (Biolegend, 423109, 1:900) was added to the cells for 30min in the dark at room temperature. The cells were washed with FACS buffer (5%FBS, 2mM EDTA in PBS) and resuspended in TruStain FcX block solution (Biolegend, 101319, 1:500) for 5min at room temperature in the dark. The primary antibody mix (CD19-APC, BD 1:300, CD45.2-PE, BD, 1:50, TCRβ-PE-Cy7, BD,1:300) was added for 30min at 4°C. The cells were then fixed and resuspended in FACS buffer to be acquired with a NovoCyte Quanteon flow cytometer (Agilent). The data was recorded with the Agilent NovoExpress software and analysed with FlowJo (Version 10.4, TreeStar). Doublets were eliminated by plotting the FSC-height and the FCS-area. The living cells were then selected. Fluorescence-Minus-One (FMO) controls were used to place the different fluorescent gates.

BMDMs. The bone marrow of the femur and tibia was flushed with Dulbecco's Modified Eagle medium (DMEM; Thermo Fisher, A1443001) using a 24G needle. The erythrocytes were removed using an ACK lysis solution. The bone marrow was frozen in freezing solution (10% DMSO in FBS) and kept for several month until further use. The thawed cells are differentiated for 10 days in DMEM supplemented with 25mM glucose (Sigma Aldrich, G8769-100ML), 4mM glutamine (Thermo Fisher, 25030081) 1mM Sodium Pyruvate (Sigma Aldrich, S8636-100ML), 10% FBS (Thermo Fisher Scientific, 10270106), 100 U/mL penicillin, 100μg/mL streptomycin (Westburg, DE17-602E) and 20ng/mL recombinant mouse macrophage colony stimulating

factor (rm M-CSF, Immunotools, 12343115). The medium was changed regularly to ensure constant supplementation of M-CSF.

Cholesterol influx/efflux. To measure cholesterol influx in inflammatory conditions, the BMDMs were stimulated in DMEM without M-CSF with 100ng/mL LPS (from E. coli O55:B5, Sigma Aldrich, L6529-1MG), 100ng/mL recombinant mouse IFNγ (Immunotools, 12343536), and 20µg/mL DiI conjugated oxLDL (Thermo Fisher Scientific, L34358) for 24h. The cells were then washed with PBS and stained with Zombie NIR (Biolegend, 423105, 1:500 in PBS) and incubated during 30min at 4°C. The cells were fixed and acquired with a NovoCyte Quanteon flow cytometer (Agilent). The data was analysed with FlowJo (Version 10.4, TreeStar). Cholesterol efflux was measured using the Cholesterol-Efflux-Assay-Kit (Merck, MAK192) according to the manufacturers protocol. The cells were stimulated with LPS and IFNγ during 24h.

Cholesterol/Triglyceride and ELISA of Liver samples.
Tissue preparation: after harvest, the livers were embedded in Tissue-Tek O.C.T. compound for cryo-sectioning. 5 cuts of 20µm each were collected for protein isolation. The cryo-cuts were lysed in lysis buffer (M-PER™ Mammalian Protein Extraction Reagent (ThermoFisher Scientific)) containing 1% protease inhibitor (Halt™ Protease Inhibitor Cocktail; ThermoFisher Scientific) and 1% phosphatase inhibitor (Halt™ Phosphatase Inhibitor Cocktail; ThermoFisher Scientific). The tissue was mechanically homogenized with a metal bead for 5min at 50Hz in the TissueLyser LT (Qiagen). Afterwards, the lysates were sonificated in a sonicator bath for 5min. The protein amount was measured with the nanodrop (Thermo Fisher Scientific), measuring the absorbance at 260nm.
Cholesterol/ triglyceride measurement: to determine cholesterol and triglyceride levels of the lysates, enzymatic assays (Roche diagnostics) were used. The cholesterol reagent was diluted 1:5 before use, otherwise the manufacturer's protocol was followed.
ELISA: inflammatory cytokines levels were measured using commercially available ELISA Kits from Thermo Fisher Scientific, according to the manufacturer’s protocol. The lysates were diluted 1:10 (TNFα) and 1:5 (IL-1β).

PI3K activity assay. Liver tissues (60 mg per animal), stored at -80 °C, were manually homogenized in a glass dounce homogenizer (Sigma-Aldrich D9063) in IP Lysis Buffer (Thermo Scientific Pierce®, Catalog No. 87787) supplemented with Halt™ Protease and Phosphatase Inhibitor Cocktail (Catalog No. 78442). For IRS2 immunoprecipitation, 1 mg of the resulting lysate was incubated overnight with 5 µg of IRS2 Polyclonal Antibody (Thermo Fisher, Catalog No. PA5-17056) in a total volume of 400 µL, following the Thermo Scientific Pierce Classic IP Kit protocol (Catalog No. 26146). PI3K activity was subsequently assessed using the Promega PI3K-Glo™ Class I Profiling Kit (Catalog No. V1690), with immunoprecipitated enzymes diluted 1:2.5 and incubated for 2 hours at room temperature before stopping the reaction. Luminescence was acquired with the SpectraMax iD3 plate reader (Molecular Devices) with an integration time of 1 sec.

Gene expression analysis of Liver samples. Total RNA was extracted from 25 mg of liver tissue, which was previously stored at −80°C, using the RNeasy Mini Kit (Qiagen, Cat. No. 74106) according to the manufacturer’s instructions. RNA quality and integrity were assessed by electrophoresis on a 1% agarose gel. One microgram of total RNA was treated with DNase I (Sigma-Aldrich, Cat. No. AMPD1) to eliminate genomic DNA contamination. Complementary

DNA (cDNA) was synthesized using the High Capacity cDNA Reverse Transcription Kit (Applied Biosystems, Cat. No. 4374966). Quantitative PCR (qPCR) was performed using SYBR Green Master Mix (Thermo Fisher Scientific, Cat. No. A46109) under standard thermal cycling conditions, including an initial denaturation at 95°C for 5 minutes, followed by 45 cycles of denaturation at 95°C, annealing at 60°C, and extension at 72°C. Relative gene expression was calculated using the comparative ΔΔCt method (5), with hypoxanthine-guanine phosphoribosyltransferase (*Hprt*) as the normalization: ΔCt was calculated by subtracting the Ct of the housekeeping gene (*Hprt)* from the Ct of the gene of interest (i.e., ΔCt = Ct_target − Ct_*Hprt*. To calculate ΔΔCt and to include error bars for the control *Ldlr*$^{-/-}$*Acod1*$^{-/-}$ averaged ΔCt values of replicates for each gene were generated. Then, for each sample, ΔΔCt was obtained by subtracting this average WT ΔCt from the sample's individual ΔCt (ΔΔCt = ΔCt_sample − ΔCt_WT_avg). For ΔΔCt analysis, all samples were compared to the control group (*Ldlr*$^{-/-}$*Acod1*$^{-/-}$).

List of primers used in this study:

Hprt: F 5′-AAGCTTGCTGGTGAAAAGGA-3′ R 5′-TTGCGCTCATCTTAGGCTTT-3′
Cpt1a, F 5′-GGTCTTCTCGGGTCGAAAGC-3′ R 5′-TCCTCCCACCAGTCACTCAC-3′
Cpt2 F 5′-CAACTCGTATACCCAAACCCAGTC-3′ R 5′-GTTCCCATCTTGATCGAGGACATC-3′
Acox1 F 5′-ACGCCACTTCCTTGCTCTTC-3′ R 5′-AGATTGGTAGAAATTGCTGCAAA-3′
Acly F 5′-GCCAGCGGGAGCACATC-3′ R 5′-CTTTGCAGGTGCCACTTCATC-3′
βactin F 5´-CAACGAGCGGTTCCGATG-´3 R 5´-GCCACAGGATTCCATACCCAA-3´
Ctp1 F 5´-ACTCCGCTCGCTCATTCCG-´3 R 5´-ACCAGTGATGATGCCATTCTTGA-´3
*Srepb*1 F 5´-GGCACTGAAGCAAAGCTGAAT-´3 R 5´-CGTCTCCACCACTTCGGGTT-´3
Acox2 F 5´-CACCCCACTGCCAGGAATAACA-´3 R 5´-CCCAAGCCTCTGGTAGGTGC-´3
Ppara F 5´-GCATTTGGGCGTATCTCACCG-´3 R 5´-CAGAGCGCTAAGCTGTGATG-´3
Pparg F 5´-CCACCAACTTCGGAATCAGCT-´3 R 5´-TTTGTGGATCCGGCAGTTAAGA-´3
Acat2 F5´-ACCAATTCCAGCCATAAAGCA-´3 R5´-GGTTTAATCCAAGTTCTTTAGCTATTGC-´3

Glucose Tolerance Test (GTT). Mice were subjected to a 6-hour fasting period with free access to water but no food. Baseline fasting blood glucose levels (mg/dL) were measured using a handheld electronic glucometer. Following this, each mouse received an intraperitoneal (i.p.) injection of glucose at a dose of 2 g/kg body weight. Blood glucose levels were subsequently monitored at 15-, 30-, 60-, and 120-minutes post-injection, using the same method.

Western Blot. Mice were fasted for 6 hours before receiving an intraperitoneal injection of insulin (Huminsulin Normal) at a dose of 0.5 U/kg. Fifteen minutes after insulin administration, the mice were euthanized, and white adipose tissue, liver, and skeletal muscle (tibialis anterior and gastrocnemius) were rapidly collected and snap-frozen in liquid nitrogen. Tissues were kept on ice until used for the preparation of protein lysates for either Western blotting or PI3-Kinase assays. For Western blot analysis, protein lysates were prepared as previously described (6). Samples were separated on 4–20% SDS-PAGE Mini-PROTEAN TGX gels (Bio-Rad Laboratories, California, USA) and transferred to 0.2 µm nitrocellulose membranes (Bio-Rad Laboratories). Membranes were blocked and then incubated with a primary antibody against phospho-AKT (#4051, Cell Signaling Technology). After washing, membranes were re-probed with an anti-AKT antibody (#9272, Cell Signaling Technology) as a loading control. Bound antibodies were detected using horseradish peroxidase-conjugated secondary antibodies and Western Lightning Plus chemiluminescence substrate (PerkinElmer, Waltham, MA, USA). Signals were visualized using the LAS-3000 imaging system (Fujifilm), and band intensities were quantified by densitometric analysis.

Single-cell RNA-seq library preparation, sequencing, and analysis. GSE247238 data generation. Cells were encapsulated in a 10x Genomics Microfluidics Chip G with barcoded oligo-dT-containing gel beads using the 10x Genomics Chromium Controller. After cleaning up these gel beads in emulsion (GEM), the cDNA was amplified, and a 3' gene expression library was constructed. The manufacturer's standards (CG000204 Rev D) were followed. The dataset is publicly available under the accession number GSE247238. Nine patient samples, nine early-stage samples and nine advanced-stage samples, are included in the final data analysis. In addition, two publicly available datasets were retrieved from the Gene Expression Omnibus (GEO) database, accession numbers GSE260657 and GSE253903. For GSE260657, carotid endarterectomy biopsies from 15 patients (7 asymptomatic, 8 symptomatic) were dissociated and single cells were captured using SmartSeq2 (~8,000 genes/cell) and then sequenced on a HiSeq 3000 (Illumina). For GSE253903, RNA was extracted from carotid plaques from 12 patients (6 asymptomatic, 6 symptomatic) using 10x Genomics Chromium Single Cell 3' v3 protocol. Samples were sequenced on an Illumina NovaSeq 6000.
The R package Seurat (version 4.1.1 for GSE247238; version 5.1.0, RRID: SCR_007322 for GSE260657 and GSE253903) in RStudio (version 1.4.1717; 4.4.0, RRID: SCR_001905) was used for scRNA-seq analysis. Genes expressed in fewer than five cells (GSE247238), and in fewer than three cells (GSE260657/GSE253903) were excluded from further analysis. For quality control, we removed low-quality cells using dataset-specific thresholds. In the GSE247238 dataset, we excluded cells with mitochondrial transcript content exceeding 15%, UMI counts greater than 20,000, and cells expressing fewer than 100 or more than 3,000 unique genes. For the GSE260657 and GSE253903 datasets, we retained only cells with 200-12,000 detected genes and mitochondrial transcript proportion below 10%.
For each dataset, normalization and variance stabilization were performed using the SCTransform function from the Seurat package, and cells were transformed into a two-dimensional map using UMAP (Uniform Manifold Approximation and Projection) with default settings, allowing the main features of each cluster to be displayed. Cell type cluster annotations were then performed. Additional differential gene expression analysis (sex- and symptom-stratified in macrophages of the GSE260657 dataset) was performed with the FindMarkers function from the Seurat package using a Poisson test on SCT counts (adjusted $p < 0.05$, absolute log2 fold change > 0.25).

Sex as biological variable. Our study examined both male and female mice and sex-dimorphic effects are reported.

Statistical analysis. Statistical differences were analysed either with the non-parametric Mann Whitney U-test, with the two-way ANOVA or with Welch's t-test in GraphPad Prism v10.2.2. Data are shown as bar graphs with single values and mean ± standard deviation (SD).

Study approval. Mouse breeding and animal experiments were performed in the animal facilities of the University of Saarland in strict accordance with the recommendations to European and German guidelines for the welfare of experimental animals. Animal experiments were approved by the Saarland state's "Landesamt für Gesundheit und Verbraucherschutz" in Saarbrücken, Germany (approval number 37/2019 and 22-2024). For experiments involving

human samples, the study was approved by the Ethics Committee of TU Munich (approval number 2799/10).

Data availability. Data are provided in the Supporting Data document online. The datasets used in this study are available on the Gene Expression Omnibus (GEO) database (ID: GSE247238, GSE260657 and GSE253903). Values for all data points in main and supplemental figures are reported in the Supporting Data Values file.

Code availability: The analysis was implemented in R (4.4.0). The code of the entire analysis workflow is available via the following Github link:
https://gitlab.com/uniluxembourg/lcsb/medical-translational-research

References:

1. Pelisek J, Hegenloh R, Bauer S, Metschl S, Pauli J, Glukha N, et al. Biobanking: Objectives, Requirements, and Future Challenges—Experiences from the Munich Vascular Biobank. *Journal of Clinical Medicine.* 2019;8(2):251.
2. Stary HC, Chandler AB, Dinsmore RE, Fuster V, Glagov S, Insull W, et al. A Definition of Advanced Types of Atherosclerotic Lesions and a Histological Classification of Atherosclerosis. *Circulation.* 1995;92(5):1355-74.
3. Redgrave JN, Gallagher P, Lovett JK, and Rothwell PM. Critical Cap Thickness and Rupture in Symptomatic Carotid Plaques. *Stroke.* 2008;39(6):1722-9.
4. Bankhead P, Loughrey MB, Fernández JA, Dombrowski Y, McArt DG, Dunne PD, et al. QuPath: Open source software for digital pathology image analysis. *Scientific Reports.* 2017;7(1):16878.
5. Bookout AL, and Mangelsdorf DJ. Quantitative real-time PCR protocol for analysis of nuclear receptor signaling pathways. *Nuclear Receptor Signaling.* 2003;1(1):nrs.01012.
6. Martus D, Williams SK, Pichi K, Mannebach-Gotz S, Kaiser N, Wardas B, et al. Cavbeta3 Contributes to the Maintenance of the Blood-Brain Barrier and Alleviates Symptoms of Experimental Autoimmune Encephalitis. *Arterioscler Thromb Vasc Biol.* 2024.

Supplemental Figures

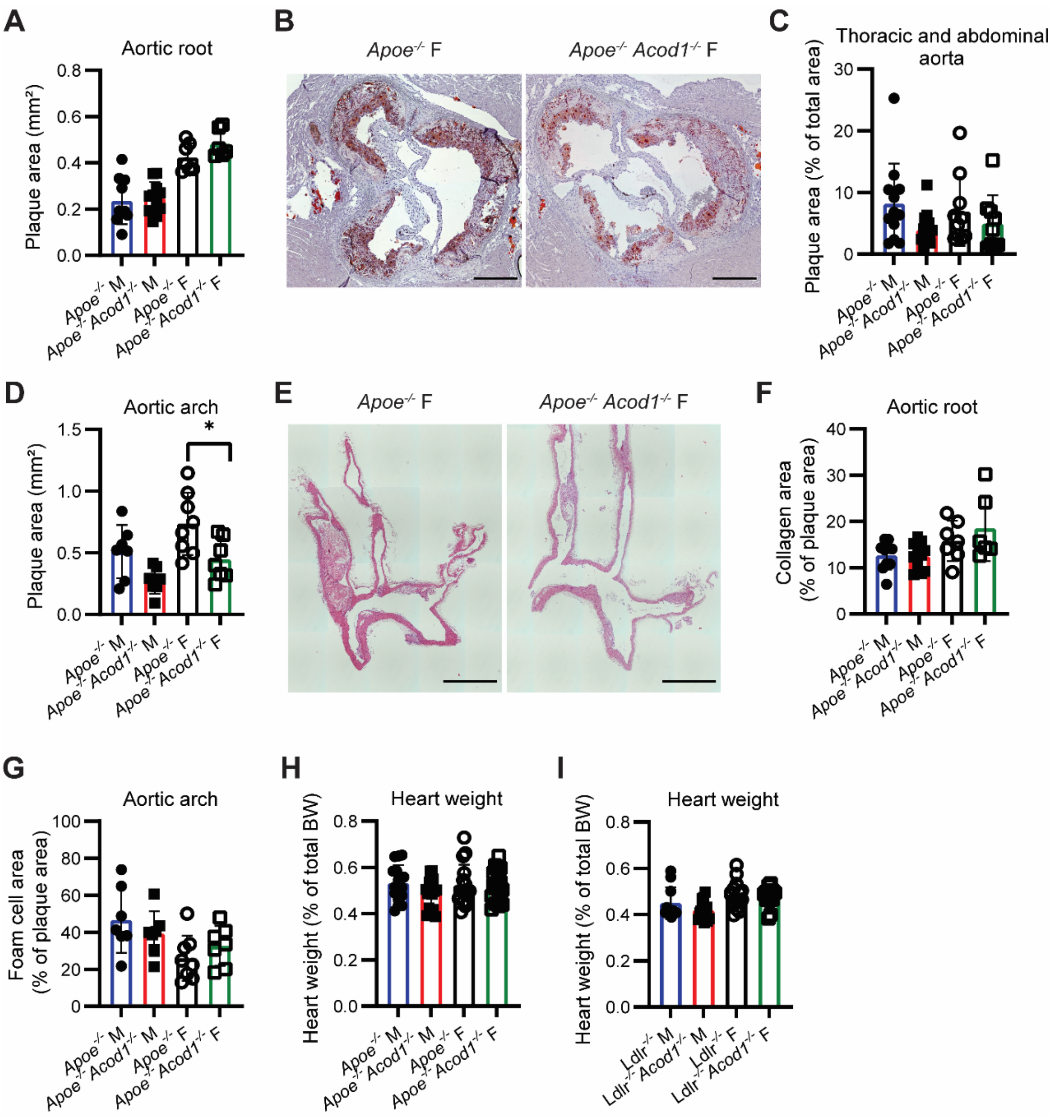

Supplemental Figure 1. Acod1 deletion has a gender dependent effect on atherosclerosis but does not affect plaque composition in the Apoe model. *Apoe*$^{-/-}$ *Acod1*$^{-/-}$ and control *Apoe*$^{-/-}$ mice were fed a western diet for 12 weeks inducing atherosclerosis. The plaque area was measured in the aortic root (**A**, males n=10-11/group, females n=6-7/group), with representative picture of the aortic root in female *Apoe*$^{-/-}$*Acod1*$^{-/-}$ and control (**B**). The plaque area was measured in the thoracic and abdominal aorta (**C**, males n=12-15/group, females n=9-10/group), and aortic arch (**D**, males n=7-8/group, females n=7-8/group). Representative images of the aortic arch in female *Apoe*$^{-/-}$*Acod1*$^{-/-}$ and control (**E**). Foam cell area was measured in the aortic arch (**F**, males and females n=7-8/group) and collagen area was measured in the aortic root (**G**, males n=10-11/group, females n=6-7/group). Heart weight of *Apoe*$^{-/-}$*Acod1*$^{-/-}$ (**H**, n=15-16/group) and *Ldlr*$^{-/-}$*Acod1*$^{-/-}$ mice (**I**, males n=11-16/group, females n=14-15/group). Results are shown as bar graphs with single values and mean ± SD. Statistical analysis were performed by Mann-Whitney test (A, C, D, F and G) or Welch's t test (H and I). *P<0.05. B: Scale bar 300µm. E: Scale bar: 1mm.

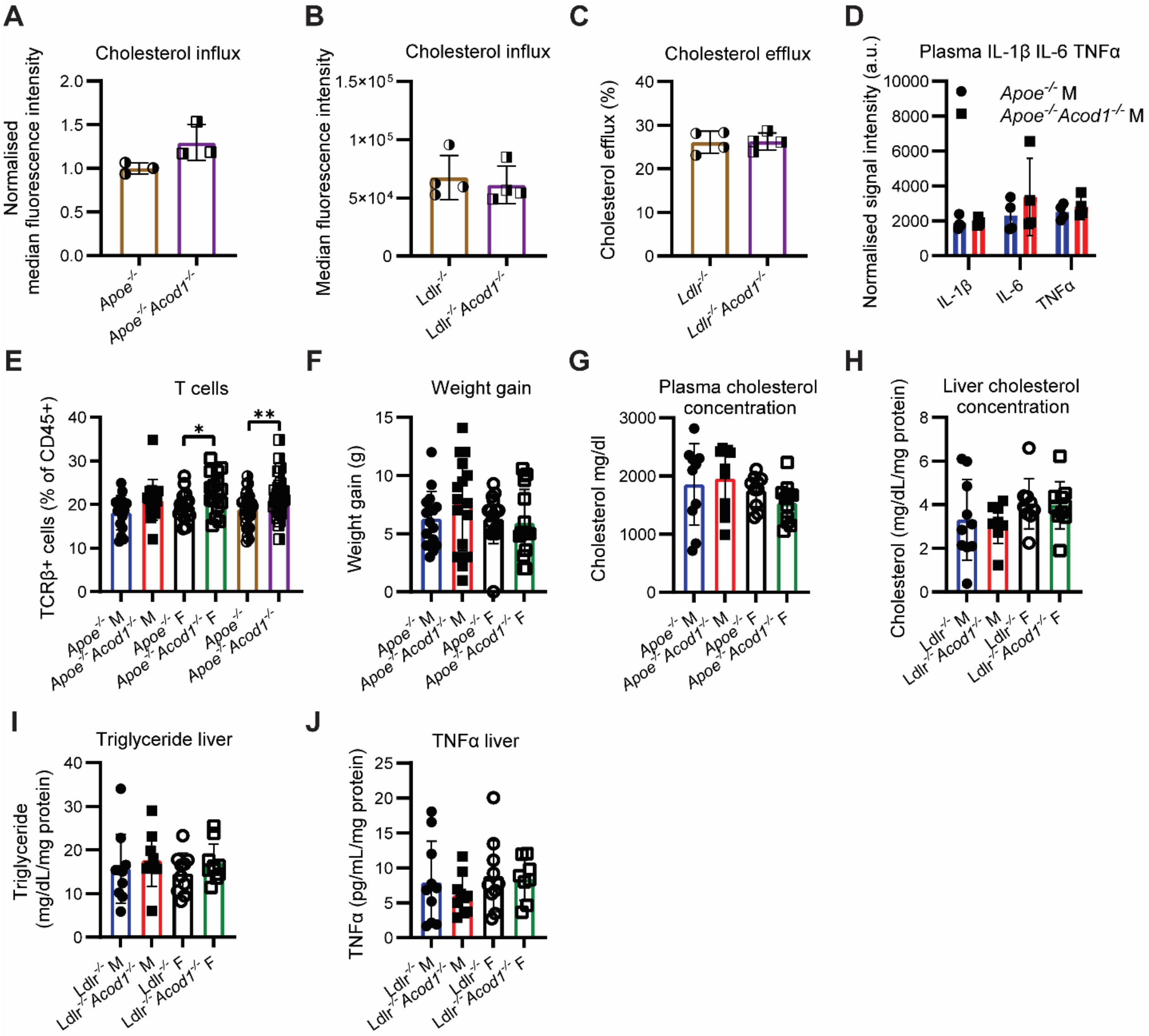

Supplemental Figure 2. Several parameters which are not affected by *Acod1* deletion in the ApoE and LDLr model. Cholesterol influx in BMDMs from *Apoe$^{-/-}$Acod1$^{-/-}$* and *Apoe$^{-/-}$* (**A**, n=3/group with 1 female and 2 males) and *Ldlr$^{-/-}$Acod1$^{-/-}$* and *Ldlr$^{-/-}$* (**B**, n=4/group with 2 females and 2 males) male and female atherosclerotic animals. BMDMs were stimulated with 100ng/mL LPS, 100ng/mL IFNγ and 20µg/mL DiI conjugated oxLDL. Cholesterol efflux of *Ldlr$^{-/-}$Acod1$^{-/-}$* and *Ldlr$^{-/-}$* BMDMs from atherosclerotic male and female animals (**C**, n=4/group with 2 females and 2 males). BMDMs were stimulated with 100ng/mL LPS and 100ng/mL IFNγ. Pro-inflammatory cytokines interleukin IL-1β, IL-6 and TNFα measured by cytokine array in the plasma of atherosclerotic male *Apoe$^{-/-}$* mice (**D**, n=4/group). Percentage of circulating T cells (gated as CD45+TCRβ+CD19- living cells) measured by flow cytometry in the blood of *Apoe$^{-/-}$* mice (**E**, male female n=16/group, pooled on the right). Weight gain of *Apoe$^{-/-}$Acod1$^{-/-}$* mice after being fed during 12 weeks a western diet (**F**, n=15-16/group). Total plasma cholesterol levels from *Apoe$^{-/-}$Acod1$^{-/-}$* and control (**G**, n=10-11/group). Cholesterol (**H**, n=8-10/group), Triglyceride (**I**, n=10/group) and TNFα (**J**, n=8-10/group) levels measured in the liver of *Acod1$^{-/-}$Ldlr$^{-/-}$* and the corresponding control mice. Results are presented as bar graphs with single values and mean ± SD. Welch's t test was used for statistical analysis. *P<0.05, **P<0.01.

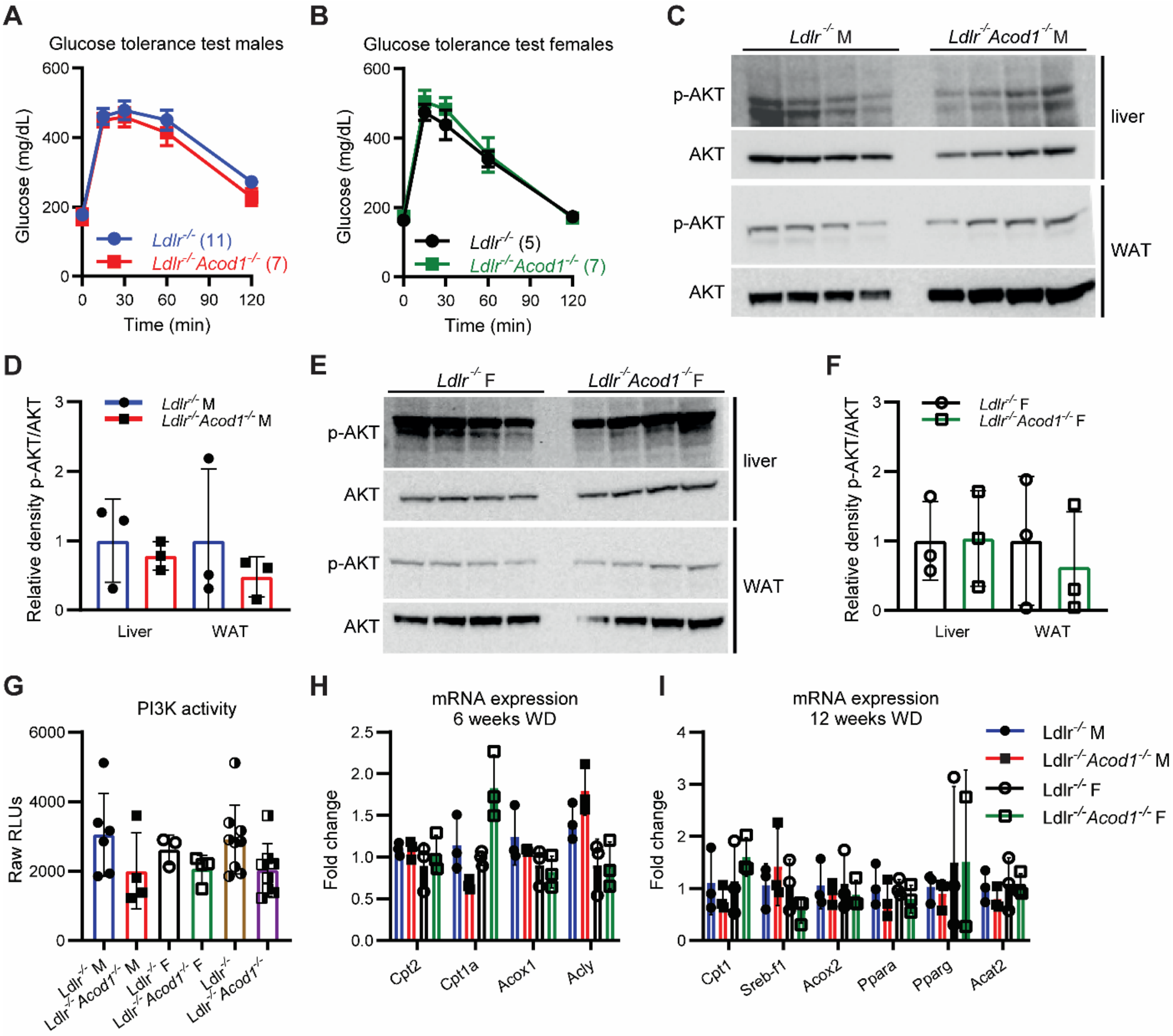

Supplemental Figure 3. Acod1 does not affect glucose clearance and insulin sensitivity. Glucose tolerance test in male (**A**, n=7-11) and female (**B**, n=5-7) *Acod1*$^{-/-}$*Ldlr*$^{-/-}$ and control mice fed during 4 weeks with a western type diet. Western blots (**C,E**) and the corresponding densitometric quantification of the antibody stain (**D,F**) showing the p-AKT/PAKT ratio obtained from the corresponding Western blots of protein lysates from the liver and white adipose tissue of *Acod1*$^{-/-}$*Ldlr*$^{-/-}$ and *Ldlr*$^{-/-}$ control male (**D**) and female (**F**) mice fed during 6 weeks with a western type diet. The tissues were collected and analyzed 15 min after insulin administration (**D,F**, n=3). PI3-Kinase activity after IRS2 pulldown from the liver of *Acod1*$^{-/-}$*Ldlr*$^{-/-}$ and *Ldlr*$^{-/-}$ control mice fed during 6 weeks with a western type diet and injected with insulin 15 min before the collection time point (**G**, n=3-6). mRNA expression of *Cpt2, Cpt1a, Acox1* and *Acly* from the liver of *Acod1*$^{-/-}$*Ldlr*$^{-/-}$ and *Ldlr*$^{-/-}$ control mice fed during 6 weeks with a western type diet and injected with insulin 15 min before the collection time point (**H**, n=3). mRNA expression of *Cpt1, Srebf1, Acox2, Ppara, Pparg and Acat2* from the liver of *Acod1*$^{-/-}$*Ldlr*$^{-/-}$ and *Ldlr*$^{-/-}$ control mice fed during 12 weeks with a western type diet (**I**, n=3). Data are shown as bar graphs with single values and mean ± SD. P-values and statistical analysis were performed by two way-ANOVA followed by Bonferroni multiple comparison test (A, B) and Student´s t-test (D, F, G, H, I).

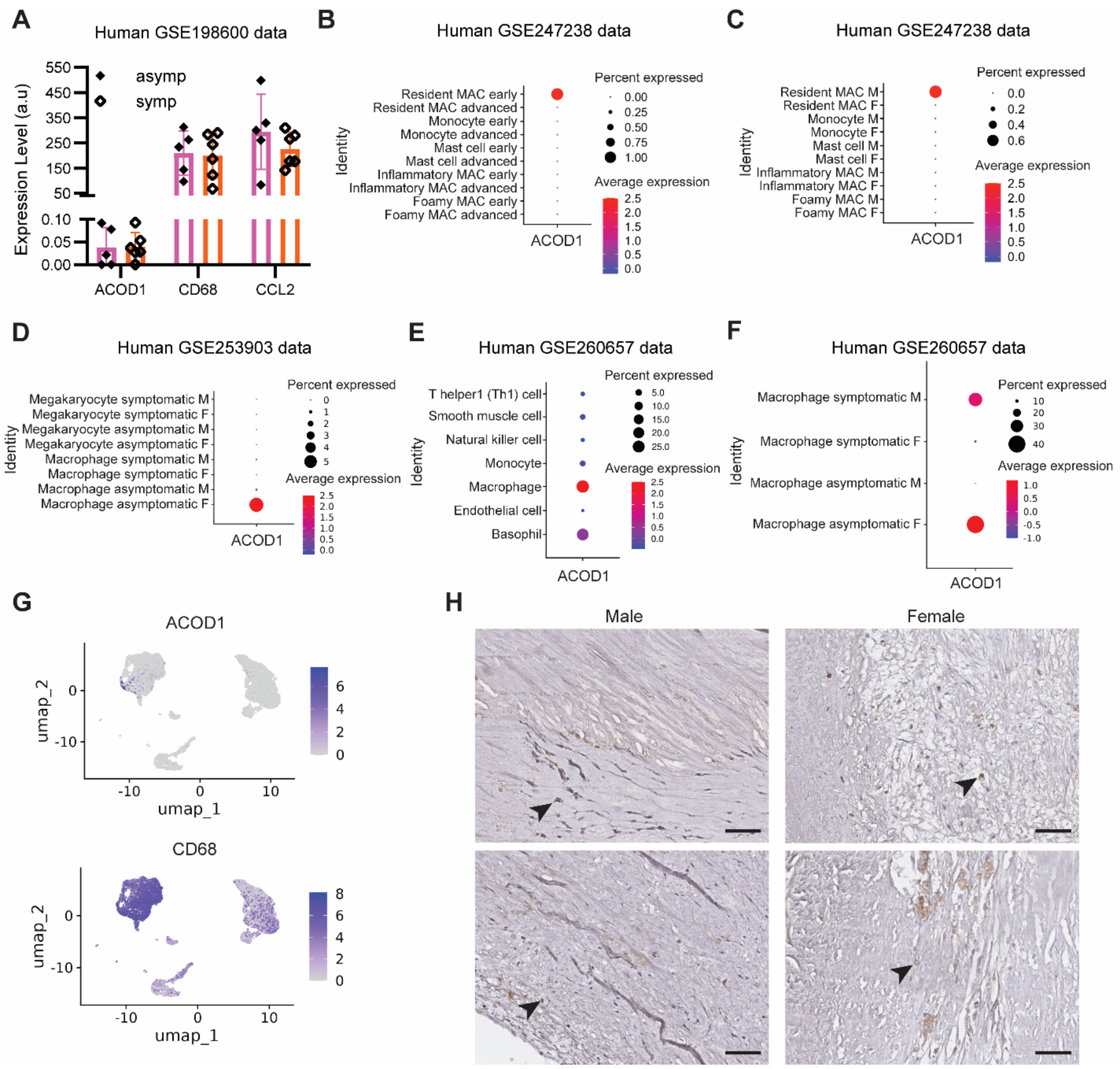

Supplemental Figure 4. ***ACOD1*** **expression is heterogenous and relatively low in human carotid plaques**. Gene expression levels of *ACOD1, CD68* and *CCL2* from a bulk RNA sequencing from carotid plaques of patients with asymptomatic (asymp) or ruptured plaques (symp) (**A**, n=5-6/group, NCBI GEO database accession GSE198600). *ACOD1* expression from single cell RNA-sequencing from early and advanced plaques (**B**) and divided by sex (**C**, NCBI GEO database accession GSE247238). *ACOD1* expression in different cell types from the transcriptomes generated of individual cells captured from atherosclerotic plaque biopsies from carotid endarterectomy from 12 patients (**D**, symptomatic male n=5, asymptomatic male n=5, symptomatic female n=1, asymptomatic female n=1, NCBI GEO database accession GSE253903). *ACOD1* expression in transcriptomes generated of individual cells captured from atherosclerotic plaque biopsies from carotid endarterectomy in various cell types (**E**) and in macrophages (**F**, symptomatic male n=5, asymptomatic male n=3, symptomatic female n=3, asymptomatic female n=4, NCBI GEO database accession GSE260657). Feature plot showing the expression of *ACOD1* and *CD68* from atherosclerotic plaque biopsies from carotid endarterectomy (**G**, NCBI GEO database accession GSE260657). ACOD1

immunohistochemical staining performed on each n=2 males (left) and females (right) sections from human advanced carotid artery plaque formation, male sample AHA classification class VII and VI, stable plaque, asymptomatic, and female class VII and VI, stable, asymptomatic (**H**, Arrowheads pointing at ACOD1 positive cells. Scale bar=50μm).
